# Apolipoprotein gene polymorphisms and plasma levels in healthy Tunisians and patients with coronary artery disease

**DOI:** 10.1186/1476-511X-7-46

**Published:** 2008-11-17

**Authors:** Raoudha Bahri, Esther Esteban, Pedro Moral, Mohsen Hassine, Khaldoun Ben Hamda, Hassen Chaabani

**Affiliations:** 1 Laboratoire de Génétique Humaine, Faculté de Pharmacie de Monastir, Université de Monastir 5000 Monastir, Tunisia; 2 Departament de Biologia Animal, Unitat d'Antropologia, Facultat de Biologia, Universitat de barcelona, Barcelona, Spain; 3 Laboratoire d'hématologie, Faculté de Pharmacie de Monastir & Hôpital Universitaire Fattouma Bourguiba, 5000 Monastir, Tunisia; 4 Département de cardiologie, Hôpital Universitaire Fattouma Bourguiba & Faculté de Médecine de Monastir 5000, Monastir, Tunisia

## Abstract

**Aim:**

To analyze apolipoprotein gene polymorphisms in the Tunisian population and to check the relation of these polymorphisms and homocysteine, lipid and apolipoprotein levels to the coronary artery disease (CAD).

**Methods:**

In healthy blood donors and in patients with CAD complicated by myocardial infarction (MI) four apolipoprotein gene polymorphisms [APO (a) PNR, APO E, APO CI and APO CII] were determined and plasma levels of total homocysteine, total cholesterol (TC), triglycerides (TG), HDL-cholesterol (HLD-C) and apolipoproteins (apo A-I, Apo B, Apo E) were measured.

**Results:**

Analysis of the four apolipoprotein gene polymorphisms shows a relative genetic homogeneity between Tunisian population and those on the other side of Mediterranean basin. Compared to controls, CAD patients have significantly higher main concentrations of TC, TG, LDL-C, apo B and homocysteine, and significantly lower ones of HDL-C, apo A-I and apo E. The four apolipoprotein gene polymorphisms have not showed any significant differences between patients and controls. However, the APO E4 allele appears to be associated to the severity of CAD and to high levels of atherogenic parameters and low level of apo E, which has very likely an anti-atherogenic role.

**Conclusion:**

Although APO (a) PNR, APO CI and APO CII genes are analyzed in only few populations, they show a frequency distribution, which is not at variance with that of APO E gene and other widely studied genetic markers. In the Tunisian population the APO E 4 appears to be only indirectly involved in the severity of CAD. In the routine practice, in addition of classic parameters, it will be useful to measure the concentration of apo E and that of Homocysteine and if possible to determine the APO E gene polymorphism.

## Introduction

Coronary artery disease (CAD) is a multifactorial disease caused by genetic and environmental factors. Apolipoprotein genes involved in lipoprotein synthesis and metabolism are considered excellent candidates for studying the susceptibility to CAD and myocardial infarction (MI) [1]. Functional variants of genes encoding lipoproteins are responsible in part for between-individual variation in the plasma levels of lipoproteins and, therefore, they are related with the risk for atherosclerosis [2].

Among apolipoproteins, apolipoprotein (a) is a highly glycosylated protein component of lipoprotein (a) [3]. It is a highly polymorphic protein whose size varies depending on the number of Kringle IV type 2 (KIV) repeats [4]. Several polymorphisms of the APO (a) gene have been described in the 5'-untranslated region [5]. A pentanucleotide repeat (PNR) polymorphism (TTTTA)n located 1.3 kb upstream of the first exon in the APO (a) gene (chromosome 6, 6q26-q27) may explain a part of the between-individual variability of lipoprotein Lp(a) [6]. However, inconsistent results have been published concerning association of this polymorphism with CAD and/or MI [7,8].

APO E gene, the most extensively studied polymorphism among apolipoproteins, has three common alleles designated E2, E3 and E4 coding for three protein isoforms (apo E2, apo E3 and apo E4, respectively) [9] that serve as a ligand for members of the low-density lipoprotein receptor (LDLR) family. This function is extremely important for the catabolism of triglyceride rich particles. It has been demonstrated that the E4 allele is associated with increased levels of plasma cholesterol, LDL-C, and increased risk for CAD [10,11].

Apolipoproteins CI and CII are constituents of very low-density and high-density lipoproteins. The genes coding for these apolipoproteins are close to the APO E gene on the long arm of chromosome 19 (19 q_13,2_). The apo CI protein inhibits the bindings of beta very low-density and intermediary density lipoproteins to lipoproteins' receptors. Apo CII is required as a cofactor in the hydrolysis of triacylglycerides of chylomicrons and VLDL by lipoprotein lipase. Two restriction polymorphisms, the HincII site in the promoter region of the APO CI gene and the AvaII site in the intron 3 of the APO CII gene [12,13], have been described in these proteins. The restriction site of APO CI has been associated with a significant increase of APO CI gene transcription [14]. Since these three genes are located in the same chromosome, the effect of the APO E, CI and CII gene cluster on serum lipid levels has been studied, particularly in Chinese Han samples of CAD patients [15,16]. The results suggest that the linkage disequilibrium between APO E and APO CI should be, in part, responsible for the development of CAD.

In the present study we analyze the polymorphism of the four apolipoprotein genes [APO (a) PNR, APO E, APO CI and APO CII] in the Tunisian population and we check the relation of these polymorphisms and homocysteine, lipid and apolipoprotein levels to CAD.

## Materials and methods

### Subjects

We conducted a hospital-based case study in 80 patients (80% men) with CAD complicated by MI (confirmed by ECG and coronary angiography) that were diagnosed and treated at the Department of Cardiology of the University Hospital Fattouma Bourguiba (Monastir, Tunisia). The mean age at diagnosis was 57.42 ± 8.37 years. Among these patients, 56 were smokers; 51 had diabetes; 35 had hypertension and 22 were hyperlipidemic. As a control group, 100 individuals (76% men with age range close to that of the patient group), free of any CAD or any related disorders, were randomly selected from the same large geographical area to which belonged the patients (the center of Tunisia). Appropriate informed consent was obtained from patients and controls in accordance with the Ethical Committee Guidelines of the participating Hospital and University.

### Plasma level measurements

Quantitative analyses of homocysteine, lipids and apolipoproteins were performed on only 50 controls and 50 patients within 24 h from collection. Plasma levels of total cholesterol (TC), triglycerides (TG) and HDL-cholesterol (HDL-C) were measured by an enzymatic colorimetric assay (using a clinical system, Beckman instruments). LDL-cholesterol concentrations were calculated by using the Friedewald formula [17]. Apolipoprotein (apo A-I, apo B, and apo E) concentrations were determined using an immunonephelometric assay on the nephelometer analyser II (Behring). The plasma concentrations of total homocysteine were measured by fluorescence polarization immunoassay on the AXSYM system.

### DNA analysis

Leukocyte DNA was extracted from blood samples by phenol-chloroform method and amplified by PCR using oligonucleotide primers and amplification conditions described previously in [6] for the PNR of APO (a), APO E, APO CI, and APO CII polymorphisms [18,12,13]. Alleles of the APO (a) PNR were identified by 10% polyacrylamide gel electrophoresis using DNA molecular weight markers. In the case of APO E, APO CI and APO CII, the PCR products were digested with Hha, HincII and AvaII enzymes, respectively and then separated by 10% polyacrylamide gel for APO E and APO CII and 2% agarose gel electrophoresis for APO CI.

### Statistical analysis

Allelic and genotypic frequencies were calculated by direct gene counting method. The chi-square test was used to test Hardy-Weinberg equilibrium of all gene variants. Differences in frequencies between cases and controls were analyzed by Fisher's exact test. Haplotype calculations for the APO E-CI-CII gene cluster were done by means of the Arlequin v 2.0 package [19]. The same package was used to estimate the comparison between our Tunisian sample and other Mediterranean populations (exact test of population differentiation). Lipid and plasma parameter measures were compared through the student's t-test. The analysis of variance (ANOVA) was used to compare the mean lipid, lipoprotein and apolipoprotein levels associated with APO E genotypes.

## Results

Allele and genotype frequencies of the APO (a) PNR, APO E, APO CI and APO CII polymorphisms analyzed in controls and patients are presented in Table 1. In all cases, genotype distributions were in Hardy-Weinberg equilibrium. APO E-APO CI-APO CII haplotypes are indicated in Table 2.

**Table 1:** Allele and genotype frequencies of APO (a) (PNR), APO E, APO CI and APO CII in control and CAD groups.

**Allele**	**Allele frequencies**		**Genotype**	**Genotype Frequencies**	
	Control (N = 100)	CAD (N = 80)		Control (N = 100)	CAD (N = 80)
	
**APO (a)**					
*7	0.145	0.168	7/7	0.020	0.020
*8	0.645	0.650	7/8	0.210	0.240
*9	0.100	0.037	8/8	0.430	0.400
*10	0.045	0.087	8/9	0.110	0.040
*11	0.065	0.056	8/10	0.040	0.100
			8/11	0.070	0.120
			9/10	0.020	0.020
			Others	0.100	0.060
P	0.276			0.182	
**APO E**					
*E2	0.045	0.037	E2/E2	-	-
*E3	0.870	0.876	E2/E3	0.080	0.080
*E4	0.085	0.087	E2/E4	0.010	-
			E3/E3	0.780	0.760
			E3/E4	0.130	0.160
			E4/E4	-	-
P	0.917			0.835	
**APO CI**					
*H1	0.915	0.893	H1/H1	0.830	0.820
*H2	0.085	0.107	H1/H2	0.170	0.180
P	1			1	
**APO CII**					
*A1	0.555	0.512	A1/A1	0.310	0.320
*A2	0.445	0.488	A1/A2	0.490	0.400
			A2/A2	0.200	0.280
P	0.618			0.635	

Alleles (*H1, *A1) and (*H2, *A2) indicate absence and presence, respectively, of the enzyme restriction site.

P: Probability values for the comparison of allele and genotype frequencies between controls and CAD.

**Table 2:** APO E - CI - CII haplotype distribution in CAD and control subjects.

**Haplotype**	**Control**	**CAD**
**E2 - H1 - A1**	0.0107	-
**E2 - H1 - A2**	-	-
**E2 - H2 - A1**	0.0342	0.0191
**E2 - H2 - A2**	-	0.0183
		
**E3 - H1 - A1**	0.4155	0.4107
**E3 - H1 - A2**	0.4216	0.4446
**E3 - H2 - A1**	0.0167	0.0196
**E3 - H2 - A2**	0.0160	-
		
**E4 - H1 - A1**	0.0596	0.03836
**E4 - H1 - A2**	0.0072	-
**E4 - H2 - A1**	0.0180	0.0247
**E4 - H2 - A2**	-	0.0244
**P**	0.922	

P: Probability values for the comparison of haplotype frequencies between controls and CAD.

### Genetic profile of the Tunisian population

APO E allele frequencies of the present control sample from a wide area of the center of Tunisia are very close to those previously described in a Tunisian sample representative of the whole country (APO E2 = 4.5, APO E3 = 85.9, APO E4 = 9.5) [20]. The lack of significant differences between Tunisian samples reveals a high degree of population homogeneity when large geographic areas are considered. In addition, our control sample does not show any significant difference with other South European populations. Data on these populations and others are presented in a previous paper conducted by our research team [20].

Allele frequencies of APO (a), APO CI, and APO CII polymorphisms in the control Tunisian sample are compared with those found in other populations (Table 3). Concerning APO (a) PNR, the allele of 8 repeats is the most common (0.635) in Tunisians agreeing with that observed in other South European populations [21-24]. However, our sample exhibits some particular features such as the lowest and highest frequencies for the alleles of 10 and 7 repeats, respectively. As a result of this particular PNR allele distribution, pairwise population comparisons reveal remarkable significant differences (p < 0.01) between our Tunisian sample and the 6 South European groups recorded in Table 3. Regarding the APO CI polymorphism, H1 is the most common allele with a frequency (0.915) similar to that found in several Spanish populations [21]. For the APO CII polymorphism, the frequencies of both alleles (0.555 for A1 and 0.445 for A2) are close to the pattern of variation observed in some Spanish groups [21]. No significant differences have been detected between Tunisians and Spaniards for these two gene polymorphisms.

**Table 3:** Distribution of APO (a), APO CI, and APO CII in Tunisian population and others Mediterranean populations.

**Population**	**N**	**Allele frequencies %**							**References**
**APO (a)**		*5	*6	*7	*8	*9	*10	*11	
Tunisia	100	0.0	0.0	14.5	64.5	10	4.5	6.5	Present study
Catalonia	88	0.0	0.6	0.6	68.2	14.2	15.9	0.6	21
Center Spain	120	0.0	0.4	0.4	76.2	12.9	8.7	1.3	21
Basque	112	0.0	0.4	0.4	69.2	18.3	11.2	0.4	21
France	199	0.2	0.2	1.0	75.9	10.5	10.7	1.5	22
Italy	218	0.0	0.0	1.1	63.0	17.0	15.0	3.9	23
Corsica	47	0.0	0.0	0.0	77.7	6.4	14.9	1.1	24
Morocco (Berbers)	138	0.4	0.0	2.9	63.8	10.9	11.6	10.5	25
**APO CI**		*H1	*H2						
Tunisia	100	91.5	8.5						Present study
Catalonia	88	84.7	15.3						21
Center Spain	120	85.4	14.6						21
Basque	111	87.4	12.6						21
Morocco (Berbers)	120	85.0	15.0						25
									
**APOCII**		*A1	*A2						
Tunisia	100	55.5	44.5						Present study
Catalonia	88	55.7	44.3						21
Center Spain	114	48.7	51.3						21
Basque	112	51.3	48.7						21
Morocco (Berbers)	117	70.1	29.9						25

N: sample size.

Alleles (*H1, *A1) and (*H2, *A2) indicate absence and presence, respectively, of the enzyme restriction site.

The pattern of allele variation in North Africa (Table 3) is represented only by a Moroccan Berber group sample [25] of ethnically Berber communities dispersed and more and less isolated in the Middle Atlas mountainous region. No significant differences have been observed between our sample and this Berber group for the APO CI polymorphism. However, for both the APO (a) PNR and the APO CII polymorphisms we have found significant differences (p = 0.010 and p = 0.031, respectively). In fact, the relatively distinctive genetic features of this Berber group beside the general genetic profile of the North African populations as that of Tunisia suggests the influence of isolation events in its genetic background.

### Comparative analyses between control and CAD groups

Lipid, lipoprotein and apolipoprotein mean concentrations are indicated in Table 4. Compared to controls, CAD patients have significantly higher values of TC, TG (triglycerides), LDL-C, apo B and homocysteine, but significantly lower values of HDL-C, apo A-I and apo E. The increased apo B levels together with the decreased apo A-I levels contribute to increase the apo B/apo A-I ratio, a quotient commonly accepted as a risk factor for CAD.

**Table 4:** Lipid and apolipoprotein mean concentrations (± SD) in controls and CAD patients.

	**Control **(N = 50)	**CAD **(N = 50)	**P**
**TC (mmol/L)**	4.01 ± 0.46	4.62 ± 0.9	<0.001
**TG (mmol/L)**	0.78 ± 1.78	1.76 ± 0.77	<0.001
**HDL-C (mmol/L)**	1.07 ± 0.12	0.92 ± 0.26	<0.001
**LDL-C (mmol/L)**	2.57 ± 0.44	2.95 ± 0.86	<0.01
**Apo AI (g/L)**	1.33 ± 0.17	1.04 ± 0.13	<0.001
**Apo B (g/L)**	0.76 ± 0.18	1.00 ± 0.35	<0.001
**Apo E (mg/L)**	50.24 ± 7.63	43.24 ± 11.33	<0.001
**Apo B/ApoAI**	0.59 ± 0.16	0.96 ± 0.31	<0.001
**Homocysteine (μmol/L)**	12.93 ± 1.12	16.6 ± 4.58	<0.001

P: Student t-test probability for comparisons among Control and CAD groups.

No significant differences have been detected between patients and controls neither for apolipoprotein gene frequencies (Table 1) nor for the haplotype distribution (Table 2) of the APO E-APO CI- APO CII gene cluster. Concerning this cluster, the linkage disequilibrium analysis showed a strong association (p < 0.0001) between APO E and APO CI in both groups and between APO E and APO CII only in controls (p = 0.008).

In spite of the lack of significant differences between patients and controls, several features are worth noting particularly at the level of APO E polymorphism. In the case of APO (a) PNR polymorphism, whereas the 8 repeats allele is found with similar frequencies in both groups, the frequency of the 9 repeats allele is higher in the control group. On the other hand, the 7 repeats allele frequency is higher in the CAD group.

As for APO E polymorphism, when the CAD sample is divided in two groups: hyperlipidemic (TC> 5.70 mmol/l) and non-hyperlipidemic subjects, the APO E4 allele is significantly (p < 0.05) higher in the hyperlipidemic patients (20.45% versus 3.51%, respectively). The same is true for the controls, although they all have normal TC concentrations (between 3 and 5 mmol/l) when controls are divided in two groups, a group with TC< 4 mmol/l and a second with TC> 4 mmol/l, the APO E4 allele is significantly (p < 0.05) higher in the second group (28.85% versus 8.33%, respectively).

Given this relation between the APO E polymorphism and the TC concentration, we have analyzed the influence of APO E genotypes on the distribution of all studied lipid, lipoprotein and apolipoprotein concentrations (Table 5). In the patients and control groups, the subjects have been regrouped according to their E2/E3, E3/E3 or E3/E4 genotypes. The E2/E4 genotype is not considered, because it has been found in only three subjects of the control group. Both in patients and controls, we have compared individuals having E2 (E2/E3) with those having E4 (E4/E3). Those having E2 show significant low levels of TC, LDL-C and apo B, whereas those having E4 show significant high values of these parameters. Apo E concentrations are also affected by APO E genotypes in the two groups. E2 carriers show high significant concentration while E3/E3 genotypes have an intermediate value and E4 carriers show a significantly low value (Table 5). Mean concentrations of other lipid parameters such as HDL-C, TG and apo A-I are not significantly different among the three APO E genotypes.

**Table 5:** Effect of APO E genotypes on plasma lipid and apolipoprotein profile in control and CAD groups.

**Genotype**	**E2/E3 **(6 controls, 4 CAD)	**E3/E3 **(25 controls, 38 CAD)	**E3/E4 **(16 controls, 8 CAD)	**P**
*TC				
Controls	3.48 ± 0.35	4.04 ± 0.49	4.20 ± 0.35	0.005
CAD	4.04 ± 1.07	4.58 ± 0.62	5.40 ± 0.67	0.007
*TG				
Controls	0.88 ± 0.10	0.73 ± 0.16	0.83 ± 0.19	0.767
CAD	1.40 ± 0.77	1.74 ± 0.70	2.11 ± 0.93	0.314
*HDL-C				
Controls	0.95 ± 0.09	1.12 ± 0.09	1.07 ± 0.13	0.074
CAD	0.86 ± 0.39	0.92 ± 0.25	0.93 ± 0.28	0.928
*LDL-C				
Controls	2.12 ± 0.38	2.56 ± 0.46	2.75 ± 0.38	0.014
CAD	2.55 ± 0.29	2.82 ± 0.70	3.77 ± 0.60	0.017
*Apo A-I				
Controls	1.33 ± 0.29	1.36 ± 0.16	1.27 ± 0.13	0.797
CAD	1.10 ± 0.12	1.03 ± 0.14	1.07 ± 0.13	0.939
*Apo B				
Controls	0.61 ± 0.06	0.73 ± 0.18	0.85 ± 0.16	0.016
CAD	0.67 ± 0.16	0.98 ± 0.36	1.23 ± 0.29	0.038
*Apo E				
Controls	57.00 ± 6.07	53.77 ± 6.59	44.62 ± 5.40	0.001
CAD	56.50 ± 20.74	42.65 ± 9.90	39.37 ± 8.76	0.043

Data is expressed as mean ± SD; P: Probability values obtained by ANOVA for the comparison between E2/E3 and E3/E4 genotype groups.

The severity of CAD in our sample has been estimated by the number (1, 2, or 3) of affected coronary vessels. Allele distributions of apolipoprotein gene polymorphisms in patients with 1, 2 and 3 affected vessels are shown in Table 6. Allele distribution comparisons between patients with 1 and 3 affected vessels have shown a significant difference (p = 0.005) only for the APO E allele comparison. This fact suggests an association between APO E polymorphisms and CAD severity. In fact, the frequency of APO E4 increases whereas the frequencies of both APO E2 and APO E3 decrease with the increased number of affected vessels.

**Table 6:** Apolipoprotein allele frequencies and CAD severity.

**Apolipoprotein alleles**	**CAD severity **(N = 80)						**P**
	1 vessel (n = 35)		2 vessels (n = 31)		3 vessels (n = 14)		
		
**APO (a) alleles**	n	(%)	n	(%)	n	(%)	0.121
7	13	18.57	11	17.74	3	10.71	
8	49	70.00	39	62.9	16	57.14	
9	2	2.86	4	6.45	0	0.00	
10	2	2.86	6	9.68	6	21.42	
11	4	5.71	2	3.23	3	10.71	
							
**APOE alleles**	n	(%)	n	(%)	n	(%)	0.005
E2	4	5.71	3	4.84	0	0.00	
E3	64	91.42	55	88.7	20	71.43	
E4	2	2.85	4	6.45	8	28.57	
							
**APOCI alleles**	n	(%)	n	(%)	n	(%)	0.285
H1	64	91.42	56	90.32	23	82.14	
H2	6	8.57	6	9.68	5	17.85	
							
**APOCII alleles**	n	(%)	n	(%)	n	(%)	1
A1	33	47.14	36	58.07	13	46.42	
A2	37	52.85	26	41.93	15	53.57	

P: probability values for the comparison of allele distribution between patients with 1 and 3 affected vessels.

## Discussion

In this study we have analyzed for the first time the polymorphisms of APO (a) PNR, APO E, APO CI and APO CII genes together with several lipid and apolipoprotein levels in Tunisian CAD patients and in controls. This constitutes an approach to the genetic characterization of the lipid profiles of Tunisian cardiovascular patients and supplies anthropological data on the Tunisian population.

Although APO (a) PNR, APO CI and APO CII gene polymorphisms have been analyzed in only few populations from South Europe (Table 2), they show frequency distribution between Mediterranean populations similar to that found according to APO E gene and other widely analyzed genetic markers [for review see [20,26-28]]. In fact, we have found modest significant differences among our Tunisian sample and those of South Europe only for the APO (a) PNR distribution. Thus, populations of the two Mediterranean shores share a relative genetic homogeneity that probably reflects a common origin and/or remarkable levels of gene flow.

Comparative analyses between control and CAD groups from Tunisia have shown important observations, particularly in comparison with those found in other populations. Concerning the analyses of plasma lipid mean concentrations, the significant high values of TC, LDL-C, apo B and TG, and the significant low values of HDL-C, apo A-I, and apo E shown by Tunisian patients with CAD have been also observed in other populations [29,30]. The homocysteine concentration, that shows mean values around 28.4% higher in CAD Tunisian patients than in controls, presents a linear relationship with the severity of CAD (14.67 μmol/L, 17.46 μmol/L, and 18.7 μmol/L for 1, 2 and 3 affected vessels respectively, p < 0.05). This relationship suggests that high homocysteine levels are associated with CAD and with its severity. Similar observations have been found in Iranian and Greek populations [31,32] but not demonstrated in a sample of American population [33]. It will be interesting to check this association in other populations and to add the quantification of homocysteine to the classic parameters analyzed in the routine practice.

In the case of the APO CI and APO CII polymorphisms, no association was noted with CAD. In fact, only few studies have analyzed the association of these polymorphisms with the disease. In a study on the Chinese population [16] the authors found that only APO CI polymorphism was associated with CAD.

Regarding the APO (a) PNR polymorphism, we ascertained a global negative association between the number of TTTTA repeats and CAD. This agrees with conclusions relating to Russian, French and Irish populations [8,34]. However, general associations between "short" TTTTA repeats (less than 10 repeats) and MI or CAD have been reported in other populations [7,23,35,36]. In addition, in this study we have noted that when the allele distribution is considered in relation to the severity of the disease (Table 6) a trend, although not significant, appears towards an increase of larger (≥ 10 repeats) alleles in the subgroup with major severity. While, in a previous study on the Japanese population, the authors showed that the homozygous genotype for the "short" 8 repeats allele was related to the number of diseased vessels [7].

The differences in results on CAD risk noted between populations probably reflect the complexity of the involvement of many genetic and environmental factors in CAD appearance. In fact, these risk factors are probably candidate factors and the appearance of the CAD does not need the presence of all of them. Namely, in a given population the presence of only some (effecter factor group) of these candidate factors, are enough for favoring the CAD appearance without or with slight secondary influence of the other factors. The effecter factor group is very likely the result of a complex gene-environmental interaction, which can vary from a population to another. Our observations relating to APO E polymorphism may illustrate this proposal.

In some populations (Italian, Turk and Iranian) a significantly higher frequency of APO E4 allele was observed in CAD patients compared with healthy subjects [10,11,30,37]. Thus, APO E4 allele can be considered among candidate risk factors for CAD. However, in our Tunisian population as in Kuwaiti, Polish and Finnish populations [38-40] no significantly higher frequency of APO E4 allele has been found in CAD patients. Namely, in these populations APO E4 allele is not among effecter factor group and it is without or with slight secondary influence on the appearance of CAD. In fact, this slight secondary influence is shown both in the Tunisian and the Finnish [40] populations by an association between the APO E4 allele and the CAD severity.

The study of the impact of APO E genotype variations on serum lipid parameters shows that CAD patients and healthy subjects with APO E4 allele have higher levels of atherogenic parameters (TC, LDL-C and apo B), whereas subjects with APO E2 allele have low levels of these parameters. Such APO E allele effects have been also noted in some other populations [11,30]. The impact of APO E allelic variation in the TC, LDL-C and apo B levels is mainly due to up and down regulation of LDL receptors by the E2 and E4 alleles respectively [41].

The effect of APO E4 on CAD severity could be the consequence of its association to significant high levels of atherogenic parameters and its association to low levels of apo E. In fact, the significant low mean concentration of apo E in CAD patients, observed also in the Italian population [10,30] suggests that apo E plays an anti-atherogenic role.

The effect of APO E genotypes on HDL-C concentration has not demonstrated the same consistency across populations. In the present study, no association has been found between APO E polymorphism and HDL-C concentrations in both patient and control groups. The relation of APO E polymorphism and HDL-C has been reported in some populations [11,37,42] but not in others [30,43]. These differences between populations can be explained by gene-environmental interaction. Indeed, HDL-C levels are affected by exercise and alcohol consumption [44,45].

## Competing interests

The authors declare that they have no competing interests.

## Authors' contributions

RB performed all the experiments and data analyses and contributed in the interpretation of results and in the editing of the manuscript, EE performed a direct help and supervision of all DNA analyses and contributed in data analyses and in the review of the manuscript, PM performed a general supervision of all DNA analyses and data analyses and participated in the review of the manuscript, MH participated in control's group collection, KBH participated in coronary artery disease patient collection, HC supervised all the work and participated in the result interpretations and in the editing and the review of the manuscript. All authors read and approved the final manuscript.
